# Activation of Bone Marrow-Derived Cells Angiotensin (Ang) II Type 1 Receptor by Ang II Promotes Atherosclerotic Plaque Vulnerability

**DOI:** 10.3390/ijms19092621

**Published:** 2018-09-04

**Authors:** Maxime Pellegrin, Karima Bouzourène, Jean-François Aubert, Aimable Nahimana, Michel A. Duchosal, Lucia Mazzolai

**Affiliations:** 1 Division of Angiology, Heart and Vessel Department, Lausanne University Hospital, 1011 Lausanne, Switzerland; karima.bouzourene@chuv.ch (K.B.); jfacgp@outlook.com (J.-F.A.); lucia.mazzolai@chuv.ch (L.M.); 2 Service and Central Laboratory of Hematology, LABORATORY and Oncology DepartmentS, Lausanne University Hospital, 1011 Lausanne, Switzerland; aimable.nahimana@chuv.ch (A.N.); michel.duchosal@chuv.ch (M.A.D.)

**Keywords:** atherosclerosis, vulnerable plaque, Angiotensin II, inflammation, 2-kidney, 1-clip ApoE^−/−^ mice, bone marrow transplantation

## Abstract

Angiotensin (Ang) II triggers vulnerable atherosclerotic plaque development. Bone marrow (BM)-derived cells are key players in atherogenesis but whether Ang II induces plaque vulnerability directly through Ang II type 1 receptor (AT1R) activation on these cells remains to be clarified. In the present study, we investigated whether a lack of AT1R on BM-derived cells might affect Ang II-mediated vulnerable plaque development. The 2-kidney, 1-clip (2K1C) model (Ang II-dependent mouse model of advanced atherosclerosis and vulnerable plaques) was generated in ApoE^−/−^ mice transplanted with AT1aR^−/−^ or AT1aR^+/+^ BM. Plasma cholesterol as well as hepatic mRNA expression levels of genes involved in cholesterol metabolism were significantly lower in 2K1C mice transplanted with AT1aR^−/−^ BM than in controls. Atherosclerotic lesions were significantly smaller in AT1aR^−/−^ BM 2K1C mice (−79% in the aortic sinus and −71% in whole aorta compared to controls). Plaques from AT1aR^−/−^ BM 2K1C mice exhibited reduced lipid core/fibrous cap and macrophage/smooth muscle cells ratios (−82% and −88%, respectively), and increased collagen content (+70%), indicating a more stable phenotype. Moreover, aortic mRNA levels of pro-inflammatory cytokines IL-12p35, IL-1β, and TNF-α were significantly reduced in AT1aR^−/−^ BM 2K1C mice. No significant differences in either the number of circulating Ly6C^high^ inflammatory monocytes and Ly6C^low^ resident anti-inflammatory monocyte subsets, or in mRNA levels of aortic M1 or M2 macrophage markers were observed between the two groups. No significant differences were observed in splenic mRNA levels of T cell subsets (Th1, Th2, Th17 and Treg) markers between the two groups. In conclusion, direct AT1R activation by Ang II on BM-derived cells promotes hepatic mRNA expression of cholesterol-metabolism-related genes and vascular mRNA expression of pro-inflammatory cytokines that may lead to plaque instability.

## 1. Introduction

Rupture of vulnerable atherosclerotic plaques is a primary underlying cause of arterial thrombosis responsible for provoking major adverse cardiovascular events such as myocardial infarction, stroke, and peripheral artery disease. Vulnerable plaque pathogenesis is complex but there is now clear evidence from both observational and experimental animal studies that the renin-angiotensin system and its final product Angiotensin II (Ang II) have significant roles in this process. Indeed, atherosclerotic renal artery stenosis resulting in stimulation of the renin-angiotensin system has been identified as a predictor of cardiovascular events in high-risk patients [1]. Moreover, we provided first experimental evidence that Ang II directly induces plaques with a vulnerable phenotype in the apolipoprotein E-deficient (ApoE^−/−^) mouse [2]. Despite the above evidence, the precise cellular and molecular mechanisms through which Ang II contributes to plaque vulnerability remain incompletely defined [3].

Most of the proatherosclerotic actions of Ang II are primarily mediated by the Ang II type 1 receptor (AT1R). Accordingly, clinical studies have demonstrated that the pharmacological blockade of AT1R reduces cardiovascular morbidity and mortality in high risk patients [4]. Both genetic disruption and pharmacological blockade of AT1R also reduce atherosclerosis severity in ApoE^−/−^ mice [5,6,7,8,9]. Similarly, we showed that AT1R blockers prevent Ang II-induced vulnerable plaque development in the 2-kidney, 1-clip (2K1C) apoE^−/−^ mouse model [10,11]. AT1R are expressed in immune cells derived from haematopoietic cells in the bone marrow (BM), including macrophages and CD4^+^ T cells which are the surrogate cells of innate and adaptive immunity, respectively, playing central roles in atherogenesis [12]. A crucial role for AT1R on BM-derived cells in mediating the pro-atherosclerotic effects of Ang II has, therefore, been suggested; however, the few existing studies using ApoE^−/−^ or low density lipoprotein receptor-deficient (LDLr^−/−^) mice infused with exogenous Ang II have yielded inconsistent results [13,14,15]. Hence, it remains unclear whether Ang II induces plaque vulnerability through direct AT1R activation on BM. Moreover, mechanisms downstream of Ang II-mediated AT1R activation promoting plaque vulnerability remain largely unknown.

The aim of the present work was to examine the effects of AT1R deficiency within BM-derived cells on Ang II-induced advanced atherosclerosis and plaque vulnerability using the 2K1C apoE^−/−^ mouse model.

## 2. Results

Irradiated ApoE^−/−^ mice were transplanted with either AT1aR^−/−^ or AT1aR^+/+^ (Wild-type) BM, and were subjected to left renal artery clipping for 4 weeks to allow development of Ang II-dependent advanced and vulnerable plaques (2K1C model). A total of *n* = 29 AT1aR^−/−^ → ApoE^−/−^ 2K1C mice and *n* = 22 AT1aR^+/+^ → ApoE^−/−^ 2K1C control mice were analyzed.

### 2.1. Physiological Parameters of Transplanted 2K1C ApoE^−/−^ Mice

As shown in Table 1, there was no significant difference in body weight (BW) gain, mean blood pressure (MBP), heart rate (HR), plasma renin activity (PRA), and plasma renin concentration (PRC) between the two groups of mice at the end of the study. Plasma total cholesterol (PTC) significantly decreased in 2K1C AT1aR^−/−^ → ApoE^−/−^ (*p* < 0.0001 versus control mice).

### 2.2. AT1aR Deficiency in BM-Derived Cells Modulates Cholesterol Metabolism 

**Figure 1:**
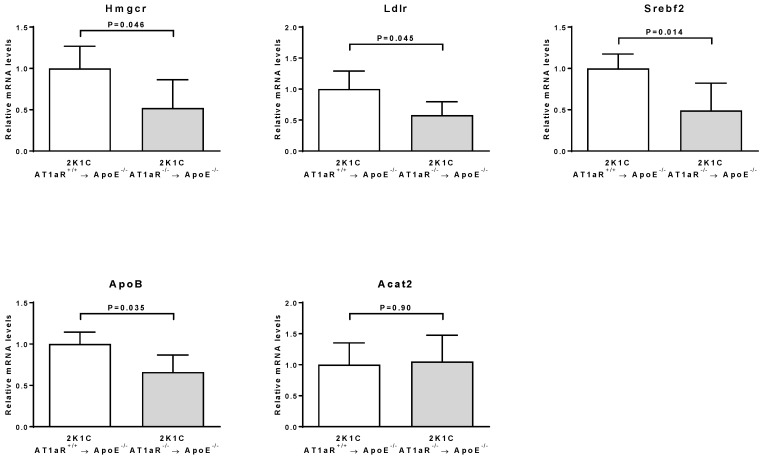
Effect of AT1aR deficiency in BM-derived cells on genes involved in cholesterol metabolism. mRNA levels of Hmgcr, Ldlr, Srebf2, ApoB, and Acat2 were determined in liver tissue by real-time RT-PCR analysis. Data were normalized to 36B4 and expressed as fold change over controls which were set at 1. *n* = 9 animals per group.

Figure 1 shows that the hepatic mRNA expression of HMG-CoA reductase (Hmgcr), LDL receptor (Ldlr), sterol regulatory element binding transcription factor 2 (Srebf2), and apolipoprotein B (ApoB) were significantly decreased in 2K1C ApoE^−/−^ transplanted with AT1aR^−/−^ BM compared to control mice. Acat2 hepatic mRNA expression was similar between the two groups (Figure 1).

### 2.3. AT1aR Deficiency in BM-Derived Cells Prevents Ang II-Induced ATS 

**Figure 2:**
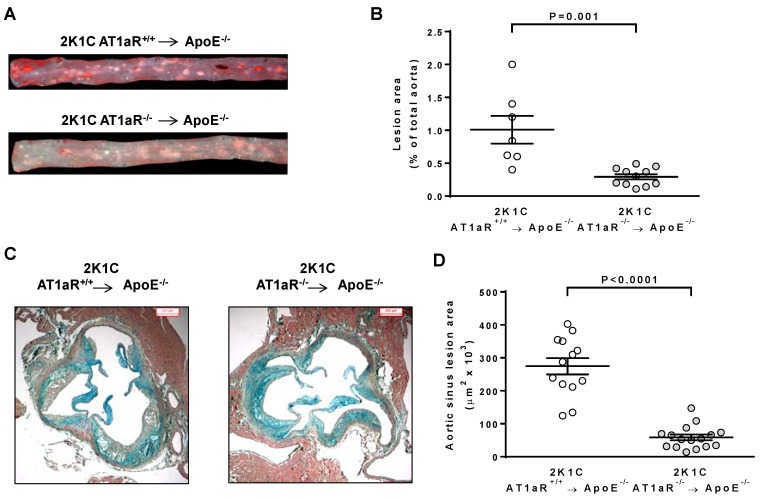
Effect of AT1aR deficiency in BM-derived cells on Ang II-mediated atherosclerosis. (**A**) Representative photographs of pinned-out aortas en face stained with Oil red O (lipid staining) from 2K1C ApoE^−/−^ mice transplanted with either AT1aR^+/+^ or AT1aR^−/−^ BM; (**B**) quantitative morphometric analysis of aortic plaque surface expressed as % of lipid deposition of the total aortic area analyzed (*n* = 7 to 11 animals per group); (**C**) representative photographs of aortic sinus cross-sections stained with Movats pentachrome (scale bar = 200 μm); (**D**) quantitative morphometric analysis of aortic sinus plaque area expressed in µm^2^ (*n* = 13 to 16 animals per group). Circles represent individual mice, horizontal lines indicate the mean, and error bars represent SEM.

Quantification of atherosclerosis in aortic preparations stained with Oil red O revealed a significant 71% decrease in atherosclerotic plaque surface in 2K1C AT1aR^−/−^ → ApoE^−/−^ mice compared to control ones (0.29 ± 0.04% versus 1.01 ± 0.21%; Figure 2A,B). Similarly, a significant 79% decrease in aortic sinus plaque surface was observed in 2K1C AT1aR^−/−^ → ApoE^−/−^ mice (58,519 ± 8664 μm^2^ versus 274,667 ± 24,859 in controls; Figure 2C,D).

### 2.4. AT1aR Deficiency in BM-Derived Cells Prevents Ang II-Mediated Plaque Vulnerability

**Figure 3:**
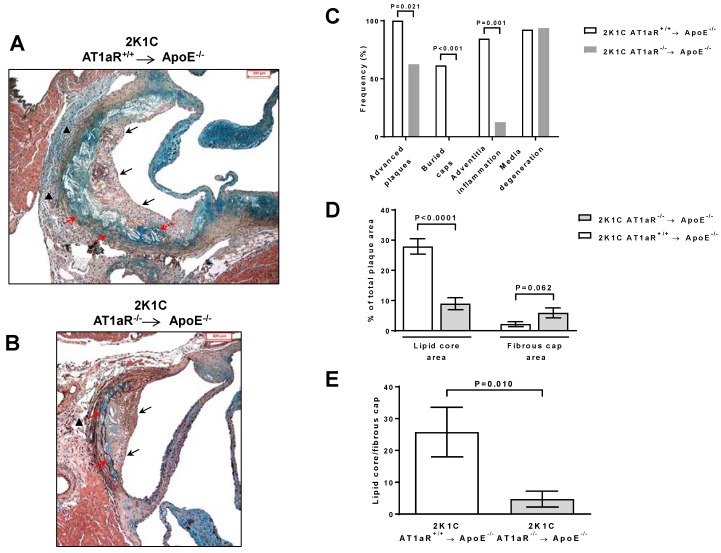
Effect of AT1aR deficiency in BM-derived cells on Ang II-mediated plaque morphology and phenotype. (**A**,**B**), Representative photographs of Movats pentachrome staining of aortic sinus cross-sections showing different plaque phenotype in 2K1C ApoE^−/−^ mice transplanted with either AT1aR^+/+^ or AT1aR^−/−^ BM (scale bar = 100 μm). Vulnerable plaque in 2K1C ApoE^−/−^ mice transplanted with AT1aR^+/+^ BM characterized by the absence of fibrous cap (black solid arrows), presence of a large lipid core (in blue) containing a vast number of cholesterol clefts, buried cap (red dotted arrow), and extensive adventitia inflammation (black triangle). Note the presence of a plaque in a coronary artery at the bottom of the photograph (**A**) Stable plaque in 2K1C ApoE^−/−^ transplanted with AT1aR^−/−^ BM demonstrating continuous and thick fibrous cap (black solid arrow), presence of small lipid core (in blue) containing a low number of cholesterol clefts, absence of buried cap and of extensive adventitia inflammation (black triangle). (**B**) Presence of media degeneration is present in plaques of both groups (red solid arrows). (**C**) Semi-quantitative analysis of markers of plaque vulnerability (the data represent the frequency of mice presenting the characteristics). (**D**) Quantitative morphometric analysis of lipid core and fibrous cap areas (Movats Pentachrome staining) over the total plaque area. (**E**) Quantification of lipid core to fibrous cap ratio. Data are mean ± SEM. *n* = 10 to 16 animals per group.

Plaque staging significantly differed among the two groups of mice. All control mice developed advanced lesions whereas these were present in 63% of 2K1C AT1aR^−/−^ → ApoE^−/−^ mice animals (*p* < 0.05; Figure 3A–C). Compared to plaques from control mice, those from 2K1C AT1aR^−/−^ → ApoE^−/−^ exhibited no buried cap, and showed reduced frequency of adventitia inflammation (*p* < 0.001, Figure 3A–C). Moreover, the lipid core area was significantly decreased by 68%, while fibrous cap area tended to increase in 2K1C AT1aR^−/−^ → ApoE^−/−^ BM mice compared to controls (lipid core area: 9.0 ± 2.0% versus 27.9 ± 2.6%, *p* < 0.0001; fibrous cap area: 5.9 ± 1.6% versus 2.2 ± 0.8%, *p* = 0.06) (Figure 3A,B,D). Overall, a significant 82% reduction in lipid core size to fibrous cap ratio was observed in 2K1C AT1aR^−/−^ → ApoE^−/−^ BM (*p* < 0.01 versus control, Figure 3E).

**Figure 4:**
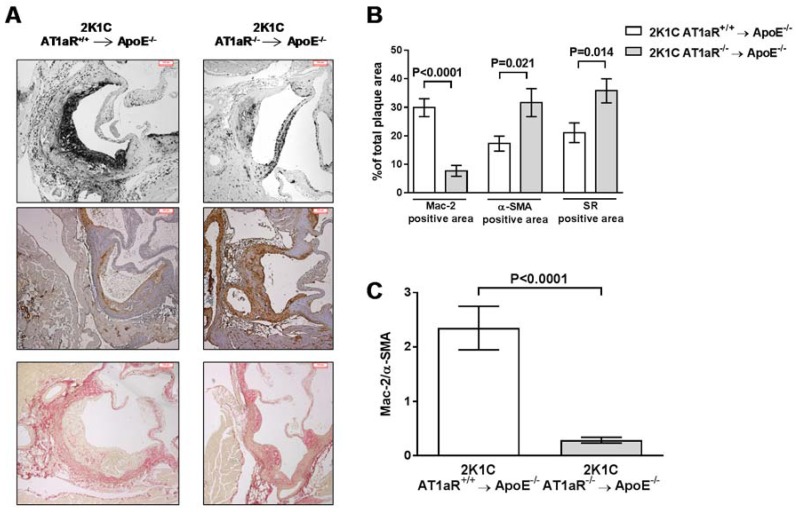
AT1aR deficiency in BM-derived cells prevents Ang II-mediated vulnerable plaques containing a high number of macrophages, and low content of SM cells and collagen. (**A**) Representative photographs of aortic sinus cross-sections immunostained for Mac-2 (top panel) and α-SM actin (middle panel) for macrophage and SM cells determination, respectively, or stained with Sirius Red (bottom panel) for collagen determination in 2K1C ApoE^−/−^ mice transplanted with either AT1aR^+/+^ or AT1aR^−/−^ BM (scale bar = 100 μm). (**B**) Quantification of Mac-2, α-SM actin, and Sirius Red positive staining over the total area of plaque. (**C**) Quantification of Mac-2 to α-SM actin ratio. Data are mean ± SEM. *n* = 12 to 16 animals per group.

Further analysis of plaque composition in 2K1C AT1aR^−/−^ → ApoE^−/−^ BM revealed a significant 74% reduction in macrophage content (7.7 ± 1.9% versus 29.9 ± 3.1%, *p* < 0.0001) and a significant 83% increase in smooth muscle (SM) cell number of (31.7 ± 4.9% versus 17.3 ± 2.6%, *p* < 0.05) accompanied by a 70% increase number of collagen fibers (35.8 ± 4.2% versus 21.1 ± 3.4%, *p* < 0.05) (Figure 4A,B). Mac-2 to α-SM actin ratio was significantly lower in 2K1C AT1aR^−/−^ → ApoE^−/−^ BM group (−88%, *p* < 0.0001; Figure 4C). 

### 2.5. AT1aR Deficiency in BM-Derived Cells Does Not Modulate Monocyte Recruitment

**Figure 5:**
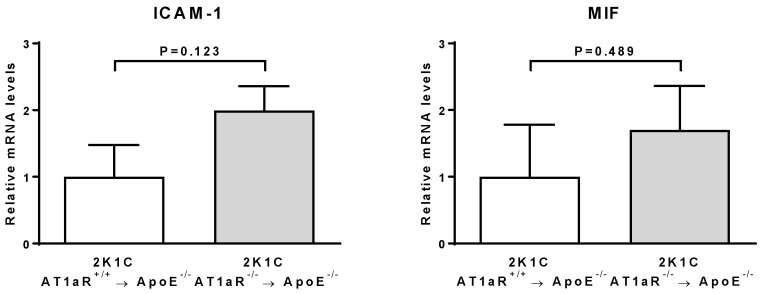
Effect of AT1aR deficiency in BM-derived cells on vascular expression of molecules involved in monocyte recruitment. mRNA levels of ICAM-1 and MIF were determined in aortic tissue by real-time RT-PCR analysis. Data were normalized to 36B4 and expressed as fold change over controls which were set at 1. Error bars are the SEM of the ∆*C*T values. *n* = 4 to 7 animals per group.

Aortic expression of intercellular adhesion molecule-1 (ICAM-1) and macrophage migration inhibitory factor (MIF) was similar between 2K1C AT1aR^−/−^ → ApoE^−/−^ BM and control mice (Figure 5). Vascular cell adhesion molecule-1 (VCAM-1) expression was not detected in any of the groups.

### 2.6. AT1aR Deficiency in BM-Derived Cells Leads to Decreased Vascular Inflammatory State

**Figure 6:**
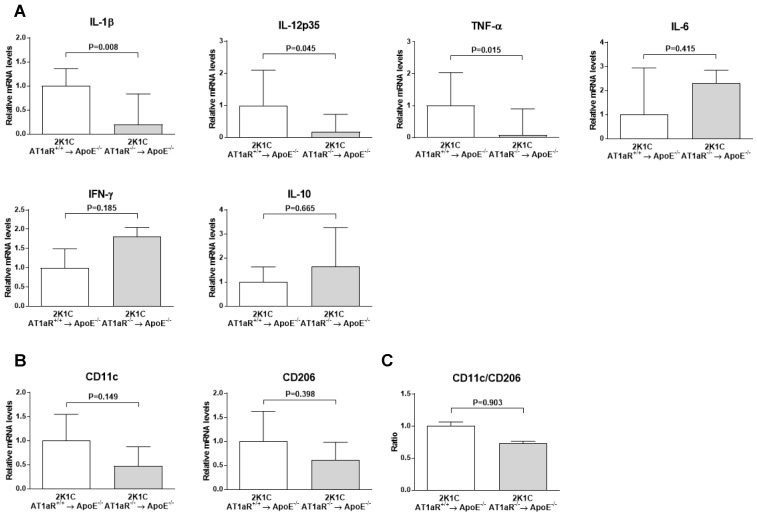
Effect of AT1aR deficiency in BM-derived cells on vascular expression of cytokines and macrophage phenotype markers. (**A**) mRNA levels of pro-inflammatory cytokines IL-1β, IL12p35, TNF-α, IL-6 and IFN-γ as well as anti-inflammatory cytokine IL-10; (**B**) mRNA levels of M1-associated marker CD11c as well as M2-associated marker CD206. mRNA levels were determined in aortic tissue by real-time RT-PCR analysis. Data were normalized to 36B4 and expressed as fold change over controls which were set at 1. Error bars are the SEM of the ∆CT values; (**C**) Quantification of CD11c to CD206 ratio (M1/M2 balance marker). *n* = 3 to 10 animals per group.

Figure 6A shows that aortic expression of pro-inflammatory cytokines interleukin (IL)-1β, IL12p35, and tumor necrosis factor (TNF)-α significantly decreased 5-fold (*p* < 0.01), 5.6-fold (*p* < 0.05), and 12.5-fold (*p* < 0.05), respectively, in 2K1C AT1aR^−/−^ → ApoE^−/−^ BM compared to controls. No significant differences in expression of pro-inflammatory interferon (IFN)-γ, and IL-6, or anti-inflammatory IL-10 cytokines were observed between the two groups. Neither CD11c M1 macrophage marker, nor CD206 M2 macrophage marker expression, nor CD11c to CD206 ratio significantly differed among the two groups (Figure 6B).

### 2.7. AT1aR Deficiency in BM-Derived Cells Does Not Affect Systemic T Helper Cells Subtypes

**Figure 7:**
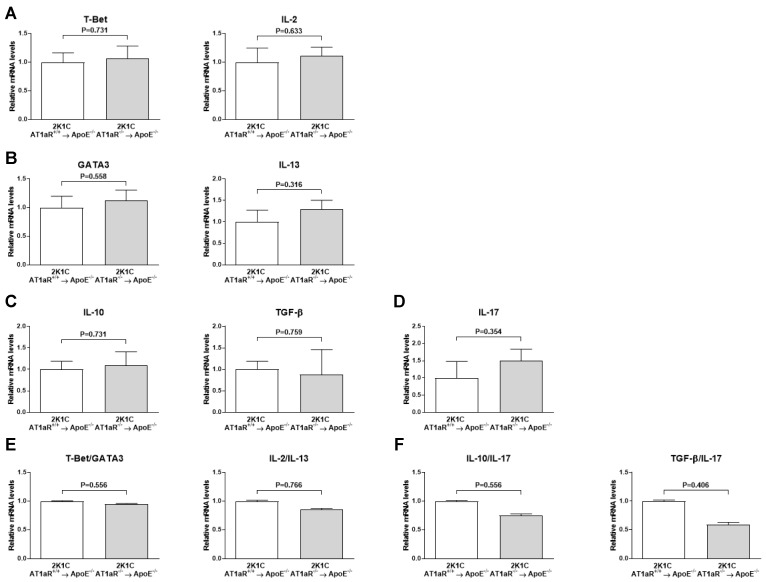
Effect of AT1aR deficiency in BM-derived cells on systemic expression of CD4^+^ T cells phenotype markers. mRNA levels of pro-inflammatory Th1 markers T-bet and IL-2 (**A**), anti-inflammatory Th2 markers GATA3 and IL-13 (**B**), Treg markers IL-10 and TGF-β (**C**), and Th17 marker IL-17 (**D**). mRNA levels were determined in splenic tissue by real-time RT-PCR analysis. Data were normalized to 36B4 and expressed as fold change over controls which were set at 1. Error bars are the SEM of the ∆CT values. Quantification of T-bet to GATA3 and IL-2 to IL-13 ratios (Th1/Th2 balance markers) (**E**), and of IL-10 to IL-17 and TGF-β to IL-17 ratios (Treg/Th17 balance markers) (**F**). *n* = 10–12 animals per group.

No statistical differences between 2K1C AT1aR^−/−^ → ApoE^−/−^ BM and control mice were observed in splenic expression of T helper type 1 (Th1) markers T-bet and IL-2 (Figure 7A); T helper type 2 (Th2) markers GATA3 and IL-13 (Figure 7B); regulatory T cells (Treg) markers IL-10 and transforming growth factor (TGF)-β (Figure 7C); and T helper type 17 (Th17) marker IL-17 (Figure 5D). Consequently, neither T-bet to GATA3 and IL-2 to IL-13 calculated ratios (Th1/Th2 balance markers) (Figure 7E), nor IL-10 to IL-17 and TGF-β to IL-17 ones (Treg/Th17 balance markers) (Figure 7F) were significantly different between groups.

### 2.8. AT1aR Deficiency in BM-Derived Cells Does Not Modulate Systemic Macrophages Phenotype

**Figure 8:**
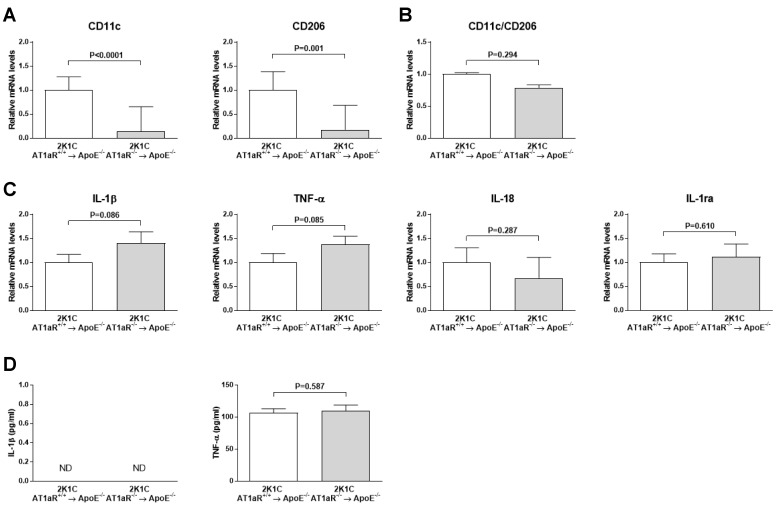
Effect of AT1aR deficiency in BM-derived cells on the systemic expression of macrophage phenotype markers. (**A**) mRNA levels of pro-inflammatory M1 macrophage marker CD11c, and anti-inflammatory M2 macrophage marker CD206. (**B**) Quantification of CD11c to CD206 ratio. (**C**) mRNA levels of M1-related pro-inflammatory cytokines IL-1β, TNF-α, IL-18 and of M2-related anti-inflammatory cytokine IL-1ra. mRNA levels were determined in splenic tissue by real-time RT-PCR analysis. Data were normalized to 36B4 and expressed as fold change over controls which were set at 1. (**D**) Plasma levels of IL-1β and TNF-α as determined by ELISA. *n* = 10–12 animals per group for real-time RT-PCR analysis. *n* = 4–8 animals per group for ELISAs; ND = Non detected.

As shown in Figure 8A, CD11c (*p* < 0.0001), and CD206 (*p* < 0.01) mRNA expression significantly similarly decreased in 2K1C AT1aR^−/−^ → ApoE^−/−^ BM (6.7-fold and 5.6-fold, respectively, versus controls), resulting in a non-significant CD11c to CD206 ratio compared to control mice (Figure 8B).

Neither M1-related cytokines IL-1β, TNF-α and IL-18, neither M2-related cytokine IL-1ra differed significantly between the two groups (Figure 8C). In accordance, ELISAs showed similar TNF-α protein concentration in plasma between the two groups while IL-1β could not be detected (Figure 8D).

### 2.9. AT1aR Deficiency in BM-Derived Cells Does Not Impact Circulating Leukocyte Profile

Percentage of circulating CD3^+^ B220^−^ T cells, CD4^+^ CD8^−^ T-helper cells, CD4^−^ CD8^+^ cytotoxic T cells, CD3^−^ B220^+^ B cells, CD11b^+^ Ly6G^+^ neutrophils, and CD11b^+^ Ly6G monocytes, subdivided into Ly6C^high^ inflammatory classical, Ly6C^med^ resident, and Ly6C^low^ non classical monocytes subsets are shown in Table 2.

Compared to controls, T cell percentage was significantly lower in 2K1C AT1aR^−/−^ → ApoE^−/−^ BM (−26%, *p* < 0.05), although no significant difference was observed in T-helper cell and cytotoxic T cell subsets. Percentage of B cells, neutrophils, and monocytes were not significantly different between the two groups. Further analysis of monocyte subsets revealed no significant difference for any of them among the two groups. Classical to nonclassical monocytes ratio was similar between the two groups (Table 2).

## 3. Discussion

Major findings presented herein are (a) Ang II drives atherosclerosis and plaque vulnerability through direct BM-derived cells activation of AT1R; (b) AT1R BM-derived cells activation leads to a reduced plasma cholesterol level that is associated with a decreased expression of genes involved in cholesterol metabolism, and to decreased aortic pro-inflammatory cytokines expression; (c) AT1R BM-derived cells activation does not modulate aortic and splenic M1 and M2 macrophage polarization, does not modulate the splenic expression pattern of Th1, Th2, Th17, and Treg markers, and finally does not impact circulating monocytes subtypes.

Macrophages and CD4^+^ T cells have been shown to play a key role in atherogenesis via several mechanisms implicating inflammation and cholesterol homeostasis among others [16]. Hence, it has been hypothesized that Ang II may induce its pro-atherosclerotic effects through direct stimulation of these immune cells AT1R. However, existing experimental studies testing this hypothesis in a mouse model of infused Ang II-induced atherosclerosis showed controversial results [13,14,15]. Two studies demonstrated that AT1R in BM-derived cells is involved in Ang II-induced atherosclerotic plaque development and destabilization [13,14] whereas the study of Koga et al. failed to observe any pro-atherosclerotic effect [15]. Conflicting reports have also been reported in hypercholesterolemic mice without Ang II stimulation [17,18,19]. To clarify the role of AT1R in Ang II-dependent atherosclerosis, we used the 2K1C ApoE^−/−^ mouse model generated in our laboratory [2]. Contrary to previous reports where the model of Ang II-dependent atherosclerosis was induced using exogenous administration of high doses of Ang II via minipumps [13,14,15], the 2K1C model is characterized by increased endogenous Ang II production in response to renal renin secretion stimulation. In the 2K1C model, all the physiological mechanisms of the renin-angiotensin-system are taken into account, as it occurs in humans. Using this model, our data showed that the absence of BM-derived cells AT1R strongly attenuates not only Ang II-induced plaque development acceleration, but also vulnerable plaque development (i.e., decreased macrophages and lipid core area, and increased SM cells, collagen, and fibrous cap area).

Several mechanisms of action may underline reduced atherosclerosis severity in 2K1C ApoE^−/−^ mice lacking AT1R in BM-derived cells. Cytokines regulate immune and non-immune cells in the vasculature thus influencing either anti- or pro-atherogenic processes [20]. We therefore tested whether the lack of AT1R on BM-derived cells affects the inflammatory state in the vascular wall of 2K1C ApoE^−/−^ mice. The present study shows significant aortic reduction in pro-inflammatory cytokines IL-1β, IL-12p35 and TNF-α in the 2K1C ApoE^−/−^ mice lacking the AT1R. Because of the increased inflammatory cells in atherosclerosis, this inevitably leads to enhanced pro-inflammatory cytokines and inversely, the decreased vascular inflammation in 2K1C mice lacking BM-derived cells AT1R could be directly related to the reduced plaque macrophage accumulation in those mice.

Adhesion molecules such as ICAM-1 and VCAM-1 as well as the cytokine MIF regulate monocyte/macrophage recruitment to the plaques [21,22]. In the present study, no significant difference in ICAM-1 and MIF vascular expression was observed in our mice (the expression of VCAM-1 was undetectable), suggesting that other mechanisms than a modulation of monocyte/macrophage recruitment may account for Ang II-AT1aR on BM-derived cells signaling-induced plaque macrophage accumulation.

Numerous studies in patients and mice have shown that a sustained cholesterol reduction is beneficial for preventing the progression of atherosclerosis [23,24]. Here, we showed that BM AT1R deficiency in 2K1C ApoE^−/−^ mice leads to a reduced plasma total cholesterol level, which may directly account for the reduced lesion formation in these mice. To explore the underlying molecular mechanisms, we examined the hepatic expression of several genes involved in cholesterol metabolism, including the rate-limiting enzyme of cholesterol synthesis (Hmgcr); Ldlr; srebf2 (the transcription factor regulating both Hmgcr and Ldlr); apoB; and Acat2, the hepatic cholesterol esterifying enzyme [25]. In agreement with our finding, expression of Hmgcr, Ldlr, Srebf2, and ApoB is lower in 2K1C mice lacking BM-derived cells AT1R. Taken together, our results strongly suggest that Ang II signaling in BM-derived cells affects cholesterol metabolism in vivo. 

In order to obtain further mechanistic insights, we explored the phenotype of vascular macrophages and circulating monocytes. To our knowledge, the present study is the first to investigate whether Ang II-activated BM-derived cells AT1R affect both lesional macrophages, and circulating monocytes activation in the setting of atherosclerosis. AT1R may directly modulate macrophage phenotype. Indeed, following pharmacological blockage of AT1R, a change in macrophage phenotype has been shown in kidney and adipose tissue in an obesity-related kidney injury model and high-fat-fed mice [26,27]. Interestingly, Yamamoto et al. recently reported decreased M1 macrophages in atherosclerotic lesions of ApoE^−/−^ mice transplanted with AT1R^−/−^ BM but with normal Ang II circulating levels [19]. Our results, showing decreased expression of pro-inflammatory cytokines in mice lacking BM-derived cells AT1R further sustain a potential polarization of vascular macrophages toward a less pro-inflammatory state. In the present study, however, we did not observe any significant differences in M1 and M2 markers expression and M1/M2 expression ratio in the aorta, despite a decrease in total macrophages plaque content in 2K1C mice lacking BM-derived cells AT1R. Differences between our study and that of Yamamoto et al. could be explained by different mouse models used. In our 2K1C mouse model, Ang II induces the development of severe and vulnerable plaques whereas Yamato et al. used ApoE^−/−^ mice with a non-stimulating renin-angiotensin system resulting in less atherosclerosis severity. In support of our findings, attenuation of murine atherosclerosis by reducing macrophages infiltration but not polarization has been shown by others [28]. 

Monocytes are key mediators in atherogenesis since they are converted in macrophages after their migration from the circulation into the intima of the arterial wall. Monocytes can also be differentiated into two distinct populations, which can be distinguished by well-characterized surface protein expression profiles. In mouse blood, classical inflammatory monocytes are identified by Ly6C^high^ expression, whereas non-classical alternative monocytes are Ly6C^low^. The literature shows that classical monocytes are precursors of M1 macrophage. In addition, studies suggest that non-classical monocytes differentiate into resident or M2 macrophages although the exact origin of M1 and M2 macrophages in atherosclerotic lesions remain to be confirmed [29]. Flow cytometry analysis showed that AT1R deficiency in 2K1C ApoE^−/−^ mice does not impact percentages of total monocytes, Ly6C^high^ monocyte subset, and Ly6C^high^ monocyte subset in the circulation. This result is in line with the results above (i.e., no change in vascular macrophage phenotype among the two groups).

Taken together, data presented herein indicate for the first time that polarization of vascular macrophages and circulating monocytes is not a primary mechanism underlying Ang II-induced plaques destabilization though AT1R on BM-derived cells.

Th1 cells have been shown to be pro-atherogenic, while Treg have anti-inflammatory and anti-atherogenic properties. The role of Th2 and Th17 remains controversial [12]. A recent study demonstrated that valsartan attenuates atherosclerosis via an increase in Th1 and Th17 and a decrease in Th2 and Treg cells in prolonged Ang II-treated ApoE^−/−^ mice [30]. One could therefore hypothesize that AT1R deficiency in BM-derived cells may shift CD4^+^ T cells polarization toward Th2- and/or Treg-oriented phenotype in our 2K1C mouse model. Gene expression analysis of Th1/Th2/Th17 and Treg signature transcription factors and/or cytokines in splenic tissue revealed no difference. The present findings are not surprising; given that Th1 and Th2 elicit M1 and M2 macrophage phenotypic polarization as explained before, and that we observed no change in macrophage polarization between our groups.

Systemic inflammation responses are considered to play a major role in the destabilization of atherosclerotic plaques [31]. Our study did not reveal significant differences in splenic expression of pro- and anti-inflammatory cytokines as well as in the plasma level of TNF-α between the two groups. Thus, we speculate that systemic inflammation does not account for the pro-atherosclerotic effect of BM-derived cells ATR1 activation by Ang II.

## 4. Materials and Methods

### 4.1. Animals and Bone Marrow Transplantation

C57BL/6 ApoE^−/−^ mice originally obtained from Charles River Laboratory (L’Arbresle, France) were used as recipients. C57BL/6 AT1aR^+/+^ (WT) and C57BL/6 AT1aR^−/−^ mice originally purchased, respectively, from The Jackson Laboratory (Bar Harbor, ME, USA) and Charles River Laboratory were used as donors. All the mice were bred and maintained in our local animal facility in individually ventilated cages with standard rodent chow and water available ad libitum. BM transplantation study was performed according to previously published protocols [13,32]. Briefly, 12–14 week-old female ApoE^−/−^ mice were lethally irradiated with a total of 900 rads using a ^137^Cs source that was delivered in two doses (4 h apart) to induce BM aplasia. On the day following irradiation, recipient mice were transplanted with BM cells freshly isolated from either AT1aR^−/−^ or AT1aR^+/+^ male mice (16–20 week-old) by injection of 1 × 10^7^ BM cells (in 200 µL of sterile PBS) per mouse into the tail vein. In brief, BM cells were isolated from pooled donor mice by flushing femur and tibia bones with ice-cold PBS-BSA 1%. Mice were maintained on antibiotic water for 1 week before BM transplantation until 2 weeks after. Successful BM transplantation was confirmed by PCR analysis of blood Sry (Y chromosome marker) DNA (Figure S1). 

Four weeks after BM constitution, mouse model of Ang II-mediated vulnerable plaque was generated in transplanted mice as described below. 

All experiments were approved by the local institutional animal committee (Service de la consommation et des affaires vétérinaires, approval number: VD2793, 16 January 2014). 

### 4.2. Mouse Model of Ang II-Mediated Vulnerable Atherosclerotic Plaques 

The renin-dependent Ang II-mediated 2K1C model was generated as originally described by our laboratory [2]. Briefly, a U-shaped stainless steel clip (0.12 mm internal diameter) was placed around the left renal artery in isoflurane-anesthetized mice, reducing renal blood flow and renal perfusion pressure. 

Four weeks after renal artery clipping (i.e., 8 weeks after BM transplantation), MBP and HR were measured from a carotid catheter as previously described [2]. Blood samples were collected from the catheter for PRA and PRC measurements as described in detail earlier [10].

### 4.3. Determination of Atherosclerotic Plaque Size by Histology

To evaluate the extent of atherosclerosis, two alternative methods were used: Analysis of atherosclerotic plaque surface en face in whole aortas and in cross-sections of the aortic sinus, as described previously [2,33]. In brief, after perfusion of the left ventricle with cold PBS, the heart and the thoracoabdominal aorta were dissected and fixed in 3.7% formalin. The aorta was then opened longitudinally, pinned on a black silicone-covered dish, and stained with Oil red O. Pictures of stained aortas were taken with a digital camera (Coolpix, Nikon, Tokyo, Japan), and the surface areas of total pinned aorta and Oil red O-stained areas were measured using computer-assisted image analysis Qwin software (Leica systems, Wetzler, Germany). Aortic plaque area was expressed as the percentage of positive Oil red O area over the total area of the aorta. 

The heart was embedded in paraffin and cross-sectioned (3 μm-thick) until reaching the aortic sinus. Sections were then stained with Movats pentachrome and photographed using a digital camera (Leica DC300F Camera, Wetzler). Pictures were then analyzed by using the Qwin software and aortic sinus plaque was expressed in µm^2^.

### 4.4. Evaluation of Atherosclerotic Plaque Staging and Phenotype by Histology and Immunohistochemical Analyses

Atherosclerotic plaque analysis was performed as previously described [2,34]. Briefly, Movats pentachrome cross-sections of the aortic sinus were used for plaque classification. Plaques were classified as intermediate (essentially composed of foam cells or with a small lipid core) or advanced (large necrotic/lipid core with multiple layers). In addition, characteristic features of plaque vulnerability were evaluated: (1) fibrous cap surface area (% of plaque surface), (2) lipid core surface area (% of plaque surface), (3) presence of media degeneration (media atrophy and/or invasion of media by plaque components), (4) presence of buried cap (defined as fibrous cap within lesions), (5) presence of adventitia inflammation (>20 polymorphonuclear cells), (6) macrophage plaque content (% of plaque surface), (7) SM cell plaque content (% of plaque surface), and (8) plaque total collagen content (% of plaque surface).

Macrophage and SM cells in plaque were identified by immunostaining with primary anti-mouse monoclonal Mac-2 and α-SM actin antibodies, respectively, followed by the appropriate biotinylated secondary antibodies. Plaque total collagen content was determined using Sirius red staining. Quantitative analysis was performed under light microscopy by means of the Qwin software.

### 4.5. Quantification of Gene Expression by Real-Time RT-PCR

In brief, total RNA was isolated from aortas and spleens using the RNeasy Fibrous Tissue Mini Kit or RNeasy Mini Kit (Qiagen, Hombrechtikon, Switzerland), respectively. Total RNA from liver was extracted using Trizol, then purified using the RNeasy MinElute Cleanup Kit (Qiagen, Hombrechtikon, Switzerland). RNA concentration and purity were measured with a spectrophotometer (Nanodrop 2000c, Thermo Scientific, Waltham, MA, USA). cDNA was then synthesized by reverse transcription using the iScriptT™ cDNA Synthesis Kit from Bio-Rad (Cressier, Switzerland). Quantitative real-time PCR was performed on a CFX96 Real-Time PCR detection system (Bio-rad, Cressier, Switzerland) in 96-well PCR plates using iQ™ SYBR^®^ Green Supermix (Bio-Rad, Cressier, Switzerland) according to the manufacturer’s protocols. The pairs of mouse primers were listed in Table S1. Data were analyzed using the comparative threshold cycles (CT) method using the housekeeping control gene 36B4 for normalization. All procedures were performed as described in previous reports [11,34]. 

### 4.6. Plasma Cholesterol and Cytokines Determination 

PTC was determined in plasma obtained by a carotid catheter at sacrifice using a commercial kit (Cholesterol FS, Diasys France). Plasma cytokines IL-1β and TNF-α were determined using mouse IL-1β/IL-1F2 and TNF-α Quantikine^®^ ELISA kits (R&D Systems Europe, Ltd., Minneapolis, MN, USA), respectively, according to the manufacturer’s instructions.

### 4.7. Analysis of Blood Leukocytes by Flow Cytometry

Blood samples obtained from the carotid catheter were processed for flow cytometry analysis according to a previously published protocol [35], with slight modifications. Briefly, after lysis of erythrocytes with a commercial lysis buffer (eBioscience, Waltham, MA, USA), cells were stained with anti-mouse CD16/CD32 antibody at 4 °C for 5 min in the dark. Then, cells were labelled with fluorochrome-conjugated antibodies at 4 °C for 10 min in the dark in two separate mixtures. Mixture 1 was used to stain T cell subsets and B cells and included the following antibodies: PE-Cy7 anti-CD3, PE anti-CD4, FITC anti-CD8, and PE-Cy5 anti-CD45R/B220. Mixture 2 was used to stain granulocytes, monocytes, and monocyte subsets (classical versus resident versus intermediate versus nonclassical), and included the following antibodies: PE anti-Ly-6G, FITC anti-Ly-6C, and PE-Cy7 anti-CD11b. Isotype-matched controls antibodies were used as negative controls. The cells were subsequently washed with 2 mL of Flow Cytometry Staining Buffer (eBiosciences) by centrifugation (5 min at 2000 rpm), and resuspended in 500 µL Flow Cytometry Staining Buffer. Samples were run on a Beckman Coulter Cytomics FC500 flow cytometer and data were analyzed with the Kaluza software (version 1.3, Beckman Coulter Indianapolis, IN, USA). A total of 50,000 events were acquired per sample. Antibodies were purchased from BD Pharmingen and Biolegend. Immune cell populations in whole blood were gated as illustrated in Figure S2.

### 4.8. Statistical Analysis

Data are expressed as mean ± SEM unless otherwise indicated. The unpaired two-tailed *t*-test was used for comparisons between the two groups. Statistical analysis of atherosclerotic plaque staging was carried out using the Fisher’s exact test. All analyses were performed using GraphPad Prism version 7.03 (GraphPad Software, La Jolla, CA, USA), and values of *p* < 0.05 were considered statistically significant.

## 5. Conclusions

In conclusion, our results provide support of a direct role of BM-derived cells AT1R in Ang II-induced atherosclerosis burden and plaque vulnerability. AT1R activation promotes hepatic mRNA expression of cholesterol-metabolism-related genes and vascular mRNA expression of pro-inflammatory cytokines. This study sheds light on mechanisms mediating the pro-atherosclerotic effect of Ang II, and might open the door to new therapeutic approaches to prevent vulnerable plaque development.

## Figures and Tables

**Table 1:** Physiological parameters in 2K1C ApoE^−/−^ mice transplanted with AT1aR^+/+^ or AT1aR^−/−^ BM. Data are expressed as mean ± SEM.

Characteristics	2K1C AT1aR^+/+^ → ApoE^−/−^	2K1C AT1aR^−/−^ → ApoE^−/−^	*p*-Value
BW gain (g)	2.26 ± 0.32	1.76 ± 0.28	0.255
MBP (mmHg)	133 ± 3	132 ± 3	0.755
HR (bpm)	602 ± 15	626 ± 15	0.269
PRA (ng/mL per h)	13.1 ± 1.4	15.1 ± 1.2	0.289
PRC (ng/mL per h)	1307 ± 169	1282 ± 155	0.913
PTC (g/L)	6.5 ± 0.15	3.5 ± 0.10	*p* < 0.0001

**Table 2:** Flow cytometry analysis of blood leukocytes in 2K1C ApoE^−/−^ mice transplanted with AT1aR^+/+^ or AT1aR^−/−^ BM mice.

Parameter	Markers	2K1C AT1aR^+/+^ → ApoE^−/−^	2K1C AT1aR^−/−^ → ApoE^−/−^	*p*-Value
T cells (% of viable cells)	CD3^+^ B220^−^	21.8 ± 1.3	16.2 ± 1.5	0.011
T-helper cells (% of T cells)	CD4^+^ CD8^−^	26.9 ± 1.0	26.6 ± 0.7	0.804
Cytotoxic T cells (% of T cells)	CD4^−^ CD8^+^	67.3 ± 1.9	70.6 ± 0.7	0.131
B cells (% of viable cells)	CD3^−^ B220^+^	15.3 ± 3.8	7.8 ± 1.1	0.082
Granulocytes (% of viable cells)	CD11b^+^ Ly6G^+^	29.3 ± 5.0	35.4 ± 3.0	0.314
Monocytes (% of viable cells)	CD11b^+^ Ly6G^−^	12.3 ± 1.9	11.0 ± 1.8	0.536
Classical monocytes (% of monocytes)	Ly6C^high^	20.7 ± 3.2	22.0 ± 2.4	0.745
Intermediate monocytes (% of monocytes)	Ly6C^medium^	21.0 ± 1.1	19.7 ± 0.5	0.322
Nonclassical monocytes (% of monocytes)	Ly6C^low^	58.3 ± 3.4	58.3 ± 2.5	0.987
Classical/nonclassical monocytes	Ly6C^high^/Ly6C^low^	0.383 ± 0.076	0.393 ± 0.054	0.917

## Supplementary Materials

The following are available online at http://www.mdpi.com/1422-0067/19/9/2621/s1, Figure S1: Assessment of BM engraftment, Table S1: Mouse primers for quantitative real-time RT-PCR, Figure S2: Flow cytometry gating strategy for analysis of cell populations in whole blood from 2K1C ApoE^−/−^ mice transplanted with AT1aR^+/+^ or AT1aR^−/−^ BM.
