# Supplementary material for: Activation of Bone Marrow-Derived Cells Angiotensin (Ang) II Type 1 Receptor by Ang II Promotes Atherosclerotic Plaque Vulnerability
Source: Int J Mol Sci. 2018 Sep 4;19(9):2621. doi: 10.3390/ijms19092621 (PMC6163751; doi:10.3390/ijms19092621)
Supplement: Supplementary file 1 [file ijms-19-02621-s001.pdf]

Supplementary: Activation of Bone Marrow-Derived Cells Angiotensin (Ang) II Type 1 Receptor by Ang II Promotes Atherosclerotic Plaque Vulnerability

Maxime Pellegrin [1,*], Karima Bouzourène [1], Jean-François Aubert [1], Aimable Nahimana [2], Michel A. Duchosal [2] and Lucia Mazzolai [1]

1 Division of Angiology, Heart and Vessel Department, Lausanne University Hospital, 1011 Lausanne, Switzerland; maxime.pellegrin@chuv.ch (M.P.); karima.bouzourene@chuv.ch (K.B.); jfacgp@outlook.com (J.-F.A.); lucia.mazzolai@chuv.ch (L.M.)

2 Service and Central Laboratory of Hematology, LABORATORY and Oncology DepartmentS, Lausanne University Hospital, 1011 Lausanne, Switzerland; aimable.nahimana@chuv.ch (A.N.); michel.duchosal@chuv.ch (M.A.D.)

* Correspondence: maxime.pellegrin@chuv.ch; Tel.: +41-21-314-07-56

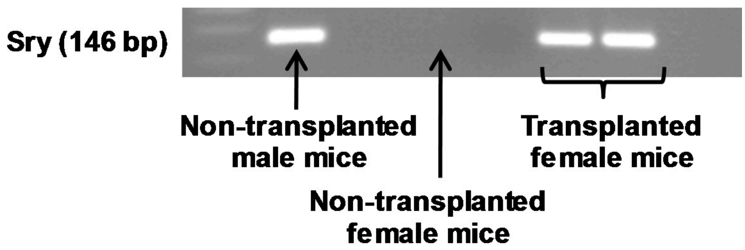

Figure S1. Assessment of BM engraftment. PCR analysis of recipient blood DNA 4 weeks after BM transplantation. PCR was carried out using Thermo Scientific Phusion Blood Direct PCR Kit. Primer sequences for mouse Sry, a marker of Y chromosome were: forward: 5′-TTCCAGGAGGCACAGAGATT-3′ and reverse: 5′-GTCCCACTGCAGAAGGTTGT-3′. PCR reactions were performed as follows: initial denaturation at 94°C for 3 min, 39 cycles with denaturation at 94°C for 30 sec, annealing at 53°C for 1 min and extension at 72°C for 2 min, followed by a final extension at 72°C for 1 min. PCR products were visualized on 2% ethidium bromide stained agarose gel. Blood from non-transplanted male mice and from non-transplanted female mice served as positive and negative controls, respectively.

Table S1. Mouse primers for quantitative real-time RT-PCR.

Gene	Forward primer	Reverse primer
IL-1β	5′-TCGCAGCAGCACATCAACAAG-3′	5′-TCCACGGGAAAGACACAGGTAG-3′
IL-12p35	5′-AGTTTGGCCAGGGTCATTCC-3′	5′-TCTCTGGCCGTCTTCACCAT-3′
TNF-α	5′-TAGCCAGGAGGGAGAACAGAAAC-3′	5′-CCAGTGAGTGAAAGGGACAGAAC-3′
IL-6	5′-GATGCTACCAAACTGGATATAATC-3′	5′-GGTCCTTAGCCACTCCTGTGTG-3′
IFN-γ	5′-TGAGACAATGAACGCTACACACTG-3′	5′-TCTTCCACATCTATGCCACTTGAG-3′
IL-10	5′-GCACTACCAAAGCCACAAAGC-3′	5′-GTCAGTAAGAGCAGGCAGCATAG-3′
CD11c	5′-ACACAGTGTGCTCCAGTATGA-3′	5′-GCCCAGGGATATGTTCACAGC-3′
CD206	5′-GTCTGTTCTGACTC GGACACTTG-3′	5′-CATGGATGTTGATGGCTACTGGAG-3′
T-bet	5′-AACCAGTATCCTGTTCCCAGC-3′	5′-TGTCGCCACTGGAAGGATAG-3′
IL-2	5′-AACCTGAAACTCCCCAGGAT-3′	5′-CATCATCGAATTGGCACTCA-3′
GATA3	5′-CAGAACCGGCCCCTTATCA-3′	5′-CATTAGCGTTCCTCCTCCAGA-3′
IL-4	5′-TCAACCCCCAGCTAGTTGTC-3′	5′-TGTTCTTCGTTGCTGTGAGG-3′
IL-13	5′-CAGCTCCCTGGTTCTCTCAC-3′	5′-CCACACTCCATACCATGCTG-3′
TGF-β	5′-CTCCCGTGGCTTCTAGTGC-3′	5′-GCCTTAGTTTGGACAGGATCTG-3′
IL-17	5′-TCCCTCTGTGATCTGGGAAG-3′	5′-CTCGACCCTGAAAGTGAAGG-3′
IL-18	5′-ACTTCTCCTGTTTGTTTGTG-3′	5′-TCTGGATACTGGGCTGTG-3′
IL-1ra	5′-ACAGTAGAAGGAGACAGAAG-3′	5′-GGTGGTAGAGCAGAAGAC-3′
VCAM-1	5′-ATTTTCTGGGGCAGGAAGTT-3′	5′-ACGTCAGAACAACCGAATCC-3′
ICAM-1	5′-AGCACCTCCCCACCTACTTT-3′	5′-AGCTTGCACGACCCTTCT-3′
MIF	5′-CCCAGAACCGCAACTACA-3′	5′-GAGCGAGGCTCAAAAGAAC-3′
Hmgcr	5′-ACGCTCATAGCTGCTGGATAG-3′	5′-AGGAAACCTTAGCCTGCTCCG-3′
Ldlr	5′-GCATCAGCTTGGACAAGGTGT-3′	5′-GGGAACAGCCACCATTGTTG-3′
Srebf2	5′-CAGACAGCCGCCCTTCAAGT-3′	5′-ATTGTGGTCAGAATGGTCCCG-3′
Acat2	5′-GAACGCATCAGGAATGAA-3′	5′-TCCCATAACAGAAGGCTCCAC-3′
ApoB	5′-GCCCATTGTGGACAAGTTGAT-3′	5′-CCAGGACTTGGAGGTCTTGGA-3′
36B4	5′-ATGGGTACAAGCGCGTCCTG-3′	5′-GCCTTGACCTTTTCAGTAAG-3′

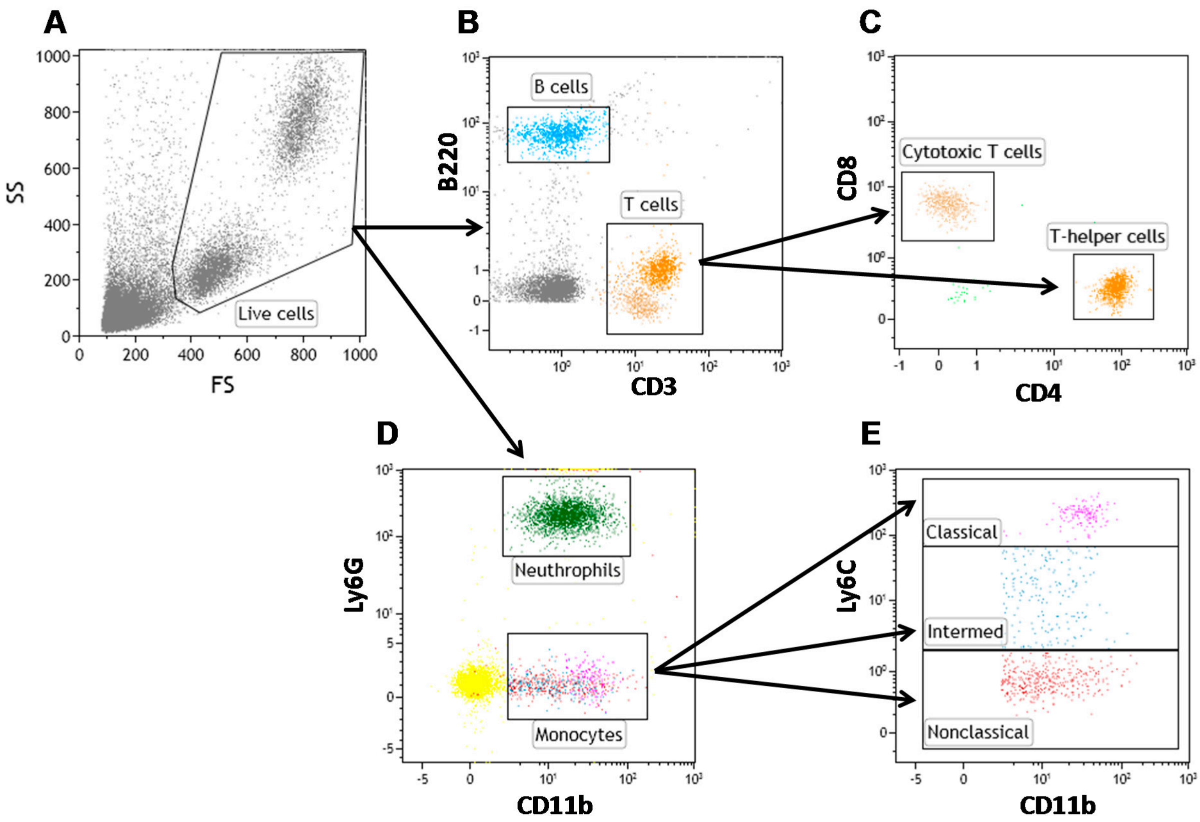

Figure S2. Flow cytometry gating strategy for analysis of cell populations in whole blood from 2K1C ApoE$^{-/-}$ mice transplanted with AT1aR$^{+/+}$ or AT1aR$^{-/-}$ BM mice. Red blood cells, dead cells, and debris were excluded based on forward scatter and side scatter (**A**). Cell surface antibodies were used to identify B and T cells (% of live cells) (**B**), T-helper cells and cytotoxic T cells (% of T cells) (**C**), neutrophils and monocytes (% of live cells) (**D**), subdivided in classical, intermediate and nonclassical (% of monocytes) (**E**).
